# Comparison of deep learning-based image segmentation methods for intravascular ultrasound on retrospective and large image cohort study

**DOI:** 10.1186/s12938-023-01171-2

**Published:** 2023-11-28

**Authors:** Liang Dong, Wei Lu, Xuzhou Lu, Xiaochang Leng, Jianping Xiang, Changling Li

**Affiliations:** 1 The Department of Cardiology, The Second Affiliated Hospital of Zhejiang University School of Medicine, Hangzhou, China; 2 ArteryFlow Technology Co., Ltd, Hangzhou, China

**Keywords:** Intravascular ultrasound, Deep learning, Image segmentation, Calcified plaque

## Abstract

**Objectives:**

The aim of this study was to investigate the generalization performance of deep learning segmentation models on a large cohort intravascular ultrasound (IVUS) image dataset over the lumen and external elastic membrane (EEM), and to assess the consistency and accuracy of automated IVUS quantitative measurement parameters.

**Methods:**

A total of 11,070 IVUS images from 113 patients and pullbacks were collected and annotated by cardiologists to train and test deep learning segmentation models. A comparison of five state of the art medical image segmentation models was performed by evaluating the segmentation of the lumen and EEM. Dice similarity coefficient (DSC), intersection over union (IoU) and Hausdorff distance (HD) were calculated for the overall and for subsets of different IVUS image categories. Further, the agreement between the IVUS quantitative measurement parameters calculated by automatic segmentation and those calculated by manual segmentation was evaluated. Finally, the segmentation performance of our model was also compared with previous studies.

**Results:**

CENet achieved the best performance in DSC (0.958 for lumen, 0.921 for EEM) and IoU (0.975 for lumen, 0.951 for EEM) among all models, while Res-UNet was the best performer in HD (0.219 for lumen, 0.178 for EEM). The mean intraclass correlation coefficient (ICC) and Bland–Altman plot demonstrated the extremely strong agreement (0.855, 95% CI 0.822–0.887) between model's automatic prediction and manual measurements.

**Conclusions:**

Deep learning models based on large cohort image datasets were capable of achieving state of the art (SOTA) results in lumen and EEM segmentation. It can be used for IVUS clinical evaluation and achieve excellent agreement with clinicians on quantitative parameter measurements.

## Introduction

Intravascular ultrasound (IVUS) is a cutting-edge medical imaging technique used in cardiology to visualize the interior of coronary artery with exceptional clarity [1]. IVUS offers a unique perspective by providing real-time cross-sectional images of the blood vessel walls, allowing physicians to obtain detailed information about the structure, composition and extent of atherosclerotic plaques. IVUS plays a very important role in improving the understanding of coronary lesions and guiding interventional treatment by accurately measuring lumen and vessel diameter as well as determining the nature and severity of plaque [2]. The different echogenic properties of the coronary vessel wall allow for a clear interpretation of the vessel structure on IVUS images. In the clinical analysis of IVUS images, accurate segmentation of the lumen and external elastic membrane (EEM) from IVUS images is of great clinical importance and helps to quantitatively assess atherosclerotic plaques by measuring lumen diameter, lumen area, plaque burden and plaque eccentricity, etc. IVUS images exhibit a variety of features, including bifurcation, side vessels, branch confluence, ultrasound artifacts, thrombosis, stent, coarctation, and various plaques, especially calcified plaques. Given this complexity of IVUS images, it can take several years to train an experienced IVUS reader. In clinical practice having a physician manually contour the lumen and EEM can be very time consuming and difficult to guarantee accuracy. Therefore, an automated method of lumen and EEM segmentation that balances speed and accuracy is urgently needed in clinical research.

Deep learning-based medical image segmentation algorithms have made remarkable progress in recent years, and new concepts and methods are constantly being introduced into the field [3–7]. Yang et al. proposed a fully convolutional network (FCN)-based IVUS-Net to segment the lumen and EEM on a dataset of 435 IVUS images including 10 cases, and achieved better results than traditional image segmentation methods [8]. This work provides a benchmark for deep learning-based methods for IVUS segmentation. Tong et al. presented an automated method for detecting lumen borders based on dictionary learning. This method required manual extraction of texture features of the image and was only used to segment the lumen [9]. Dong et al. proposed an 8-layer U-Net network based on segmentation of the lumen and EEM, but the sample size was too small and no further generalization performance was verified on multiple IVUS images [10]. In contrast, the study by Du et al. remedied these shortcomings. They constructed a multicenter, IVUS dataset containing 6516 images, synthetically measured multiple convolutional segmentation networks, and validated the generalization performance on a variety of IVUS images [11]. Combining the above related studies, we believe it is necessary to measure the segmentation effect of IVUS lumen and EEM on a larger dataset using the latest models in the field of image segmentation.

In this study, we constructed IVUS image dataset containing over 11,000 images with diverse features and categories. Second, we comprehensively compared the segmentation performance of the latest image segmentation models on lumen and EEM based on recent model advances in the field of image segmentation, and performed diversity generalization performance tests. Further, we compared the agreement of relevant quantitative clinical parameters with manual segmentation computations based on the best segmentation results.

## Results

### Demographics of the study cohort

We performed a demographic analysis of 11,070 IVUS images of all 113 cases on the full study cohort and analyzed the statistical differences between them in the Table 1. There were no significant differences between the cohorts in terms of the proportion of male patients, age and the proportion of patients with CAD, which also suggests that the internal distribution in our randomly divided data cohort is reasonable. In terms of the coronary vessels involved, although the vast majority of lesions were concentrated in the left anterior descending (LAD) (65.49%), the distribution of lesion cases across vessels was relatively even in three study cohorts. In terms of IVUS image categories, we divided all images into seven categories: calcified plaque, bifurcation, adjacent vessels, stents, guidewire artifacts, lipid fibrous plaque and normal vessels (None), with a relatively large proportion of calcified plaque, guidewire artifacts and lipid fibrous plaque, but with little difference in overall distribution.

**Table 1:** Overview of the baseline characteristics of all study cohort

	Training cohort, *n* = 79	Validation cohort, *n* = 11	Testing cohort, *n* = 23	*P*-value
Patients with CAD, *n* (%)	78 (98.73)	10 (90.91)	22 (95.65)	0.27
Males, *n* (%)	45 (56.96)	7(63.64)	15 (65.22)	0.74
Age, mean ± SD	68.46 ± 8.86	73.0 ± 3.56	69.0 ± 4.44	0.40
Age > 60, *n* (%)	30 (37.98)	3 (27.27)	6 (26.09)	0.50
Involved vessel				
LAD, *n* (%)	50 (63.29)	8 (72.73)	16 (69.56)	0.74
LCX, *n* (%)	7 (8.86)	0 (0)	2 (8.70)	0.59
RCA, *n* (%)	13 (16.46)	3 (27.27)	5 (21.74)	0.63
Pullbacks	79	11	23	< 0.001
Images	7808	1073	2189	< 0.001
IVUS image categories				
Calcified plaque, *n* (%)	2425 (31.06)	393 (36.63)	651 (29.74)	< 0.001
Bifurcation, *n* (%)	338 (4.33)	55 (5.13)	145 (6.62)	< 0.001
Adjacent vessels, *n* (%)	916 (11.73)	199 (18.55)	197 (9.00)	< 0.001
Stent, *n* (%)	171 (2.19)	62 (5.78)	60 (2.74)	< 0.001
Guidewire artifacts, *n* (%)	2518 (32.25)	223 (20.78)	945 (43.17)	< 0.001
Lipid fibrous plaque, *n* (%)	3962 (50.74)	467 (43.52)	1109 (50.67)	< 0.001
None, *n* (%)	1411 (18.07)	203 (18.92)	422 (19.28)	0.39

Analysis of variance (ANOVA) was used as the test for the difference of mean for continuous variables such as Age, Pullbacks and Images. Chi-squared test was used to test for the difference of proportion for categorical variables such as Patient with CAD, Males, Age > 60, Involved vessel and IVUS image category. Multiple categories may exist for the same IVUS image

*CAD* coronary artery disease, *LAD* left anterior descending, *LCX* left circumflex, *RCA* right coronary artery

### Performance of different models

**Fig. 1:**
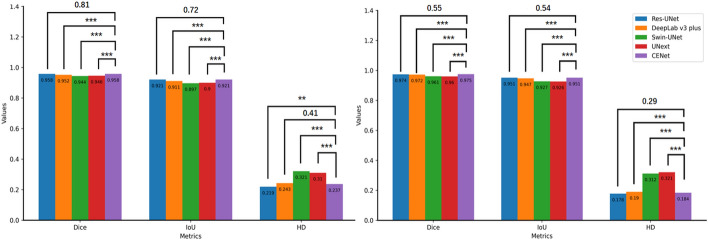
Comparison of the performance of the 5 segmentation models for lumen (left subplot) and EEM (right subplot). ANOVA was used to statistically analyze the variability in the performance of the different models. *0.01 ≤ *P* < 0.05; **0.001 ≤ *P* < 0.01; ***P < 0.001

Table 2 shows the segmentation performance of the five segmentation models for the lumen and EEM on the test set images. The segmentation metrics (mean ± standard deviation) include dice similarity coefficients (DSC), intersection over union (IoU) and Hausdorff distance (HD). Among them CENet achieved the highest in both DSC and IoU for the lumen and EEM segmentation. Res-UNet, on the other hand, performed best in HD, with HD of 0.219 and 0.178 for the lumen and EEM, respectively. Figure 1 further showed the variability between the segmentation performance of different models in terms of statistical tests.

**Table 2:** Performance of the five segmentation models on the testing cohort for lumen and EEM segmentation with metrics including dice similarity coefficient (DSC), intersection over union (IoU) and Hausdorff distance (HD)

Models	Lumen			EEM			Parameters (M)
	DSC	IoU	HD (mm)	DSC	IoU	HD (mm)	
Res-UNet	0.958 ± 0.037	0.921 ± 0.058	**0.219 ± 0.210**	0.974 ± 0.024	0.951 ± 0.043	**0.178 ± 0.200**	24.45
DeepLab v3 plus	0.952 ± 0.040	0.911 ± 0.064	0.243 ± 0.226	0.972 ± 0.027	0.947 ± 0.046	0.190 ± 0.192	54.11
Swin-UNet	0.944 ± 0.045	0.897 ± 0.070	0.321 ± 0.242	0.961 ± 0.037	0.927 ± 0.062	0.312 ± 0.231	41.39
UNeXt	0.946 ± 0.039	0.900 ± 0.064	0.310 ± 0.276	0.960 ± 0.046	0.926 ± 0.073	0.321 ± 0.330	1.47
CENet	**0.958 ± 0.035**	**0.921 ± 0.057**	0.237 ± 0.223	**0.975 ± 0.024**	**0.951 ± 0.042**	0.184 ± 0.197	29.00

The number of parameters representing the size of the model was also listed

The bold values indicate the optimal values for different models at the current metrics

**Fig. 2:**
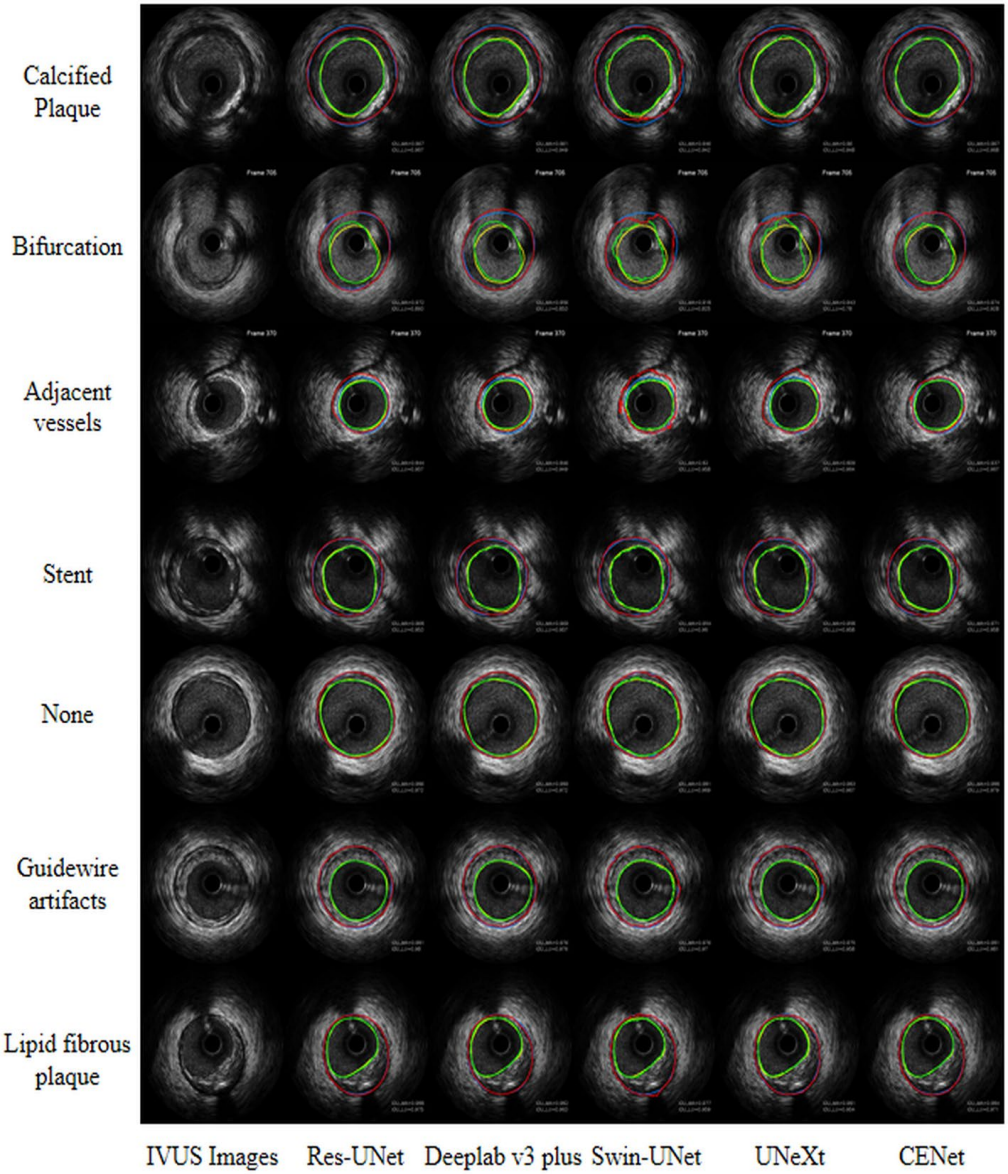
Visualization of the lumen and EEM contours of the 5 segmentation models on the 7 IVUS image categories. The yellow, blue, green, and red contours correspond to the manually delineated lumen border, the manually delineated EEM border, the model segmented lumen border, and the model segmented EEM border, respectively

In Table 3, we further compared the segmentation performance of the five models on seven different subsets of the IVUS categories in the testing cohort, including calcified plaque, bifurcation, adjacent vessels, stent, guidewire artifacts, lipid fibrous plaques and normal images (None). There was crossover between subsets, meaning that a single IVUS image may have several IVUS image categories. The results in Table 3 can be used as a further breakdown of Table 2, and thus the performance comparison between the models in Table 3 is consistent with Table 2. Both Res-UNet and CENet performed very robustly on each subset. Overall, Res-UNet performed relatively better on lumen segmentation for each subset, while CENet was better on EEM. Figure 2 exhibited the visualization of the segmentation contours of the 5 models on different IVUS image categories.

**Table 3:** Means and standard deviations of the dice similarity coefficient (DSC), intersection over union (IoU) and Hausdorff distance (HD) of five segmentation models evaluated on seven different IVUS categories subsets of the testing cohort

	Models	Lumen			EEM		
		DSC	IoU	HD (mm)	DSC	IoU	HD (mm)
Calcified plaque (651 images)	R	**0.961 ± 0.026**	**0.925 ± 0.045**	**0.202 ± 0.188**	0.963 ± 0.030	0.930 ± 0.051	**0.257 ± 0.228**
	D	0.955 ± 0.032	0.915 ± 0.053	0.227 ± 0.230	0.962 ± 0.027	0.929 ± 0.047	0.259 ± 0.215
	S	0.947 ± 0.032	0.902 ± 0.054	0.303 ± 0.210	0.951 ± 0.034	0.908 ± 0.058	0.379 ± 0.233
	U	0.950 ± 0.032	0.906 ± 0.054	0.294 ± 0.294	0.950 ± 0.040	0.907 ± 0.067	0.414 ± 0.373
	C	0.960 ± 0.024	0.924 ± 0.043	0.222 ± 0.208	**0.964 ± 0.028**	**0.932 ± 0.049**	0.262 ± 0.246
Bifurcation (145 image ± s)	R	0.953 ± 0.044	0.913 ± 0.071	**0.274 ± 0.325**	**0.969 ± 0.031**	0.942 ± 0.053	**0.267 ± 0.403**
	D	0.947 ± 0.047	0.903 ± 0.075	0.318 ± 0.413	0.968 ± 0.030	0.939 ± 0.051	0.281 ± 0.407
	S	0.941 ± 0.041	0.891 ± 0.067	0.393 ± 0.328	0.955 ± 0.038	0.916 ± 0.063	0.401 ± 0.311
	U	0.933 ± 0.049	0.879 ± 0.080	0.473 ± 0.518	0.953 ± 0.045	0.913 ± 0.073	0.585 ± 0.724
	C	**0.955 ± 0.041**	**0.917 ± 0.068**	0.294 ± 0.387	0.969 ± 0.035	**0.943 ± 0.058**	0.271 ± 0.410
Adjacent vessels (197 images)	R	0.962 ± 0.027	0.928 ± 0.046	**0.161 ± 0.112**	0.971 ± 0.031	0.945 ± 0.054	**0.160 ± 0.181**
	D	0.956 ± 0.028	0.917 ± 0.048	0.187 ± 0.129	0.963 ± 0.049	0.932 ± 0.077	0.220 ± 0.255
	S	0.944 ± 0.043	0.897 ± 0.067	0.277 ± 0.165	0.940 ± 0.065	0.892 ± 0.101	0.398 ± 0.312
	U	0.947 ± 0.037	0.901 ± 0.061	0.297 ± 0.342	0.928 ± 0.086	0.876 ± 0.129	0.552 ± 0.605
	C	**0.962 ± 0.024**	**0.928 ± 0.042**	0.168 ± 0.109	**0.973 ± 0.024**	**0.949 ± 0.042**	0.194 ± 0.298
Stent (60 images)	R	**0.957 ± 0.020**	**0.918 ± 0.036**	**0.241 ± 0.165**	0.968 ± 0.020	0.939 ± 0.036	**0.229 ± 0.147**
	D	0.948 ± 0.022	0.902 ± 0.039	0.270 ± 0.150	0.968 ± 0.019	0.939 ± 0.034	0.234 ± 0.151
	S	0.936 ± 0.036	0.881 ± 0.061	0.381 ± 0.208	0.956 ± 0.024	0.917 ± 0.043	0.380 ± 0.215
	U	0.945 ± 0.027	0.897 ± 0.047	0.33 ± 0.188	0.951 ± 0.038	0.908 ± 0.066	0.407 ± 0.295
	C	0.956 ± 0.018	0.916 ± 0.032	0.251 ± 0.159	**0.968 ± 0.017**	**0.939 ± 0.032**	0.244 ± 0.141
Guidewire artifacts (945 images)	R	0.953 ± 0.047	0.913 ± 0.720	**0.252 ± 0.243**	0.976 ± 0.022	0.954 ± 0.039	**0.183 ± 0.203**
	D	0.949 ± 0.045	0.906 ± 0.071	0.268 ± 0.248	0.975 ± 0.020 ±	0.952 ± 0.036	0.186 ± 0.192
	S	0.943 ± 0.041	0.895 ± 0.066	0.334 ± 0.220	0.965 ± 0.032	0.933 ± 0.053	0.306 ± 0.220
	U	0.942 ± 0.044	0.894 ± 0.069	0.346 ± 0.301	0.967 ± 0.029	0.937 ± 0.049	0.313 ± 0.338
	C	**0.954 ± 0.043**	**0.914 ± 0.067**	0.273 ± 0.264	**0.977 ± 0.021**	**0.955 ± 0.038**	0.192 ± 0.231
None (422 images)	R	**0.966 ± 0.019**	**0.934 ± 0.035**	**0.180 ± 0.125**	0.976 ± 0.025	0.954 ± 0.043	**0.138 ± 0.136**
	D	0.961 ± 0.023	0.926 ± 0.041	0.202 ± 0.146	0.970 ± 0.041	0.945 ± 0.066	0.177 ± 0.202
	S	0.953 ± 0.033	0.912 ± 0.053	0.265 ± 0.162	0.957 ± 0.052	0.922 ± 0.083	0.298 ± 0.233
	U	0.956 ± 0.023	0.917 ± 0.041	0.260 ± 0.206	0.950 ± 0.069	0.912 ± 0.107	0.331 ± 0.362
	C	0.964 ± 0.021	0.932 ± 0.038	0.202 ± 0.154	**0.977 ± 0.023**	**0.955 ± 0.040**	0.160 ± 0.204
Lipid fibrous plaques (1109 images)	R	0.954 ± 0.046	0.914 ± 0.070	**0.244 ± 0.243**	**0.980 ± 0.018**	**0.961 ± 0.032**	**0.144 ± 0.175**
	D	0.947 ± 0.048	0.902 ± 0.074	0.268 ± 0.245	0.978 ± 0.017	0.958 ± 0.031	0.155 ± 0.162
	S	0.939 ± 0.054	0.889 ± 0.081	0.354 ± 0.277	0.968 ± 0.030	0.940 ± 0.050	0.278 ± 0.222
	U	0.940 ± 0.046	0.890 ± 0.073	0.338 ± 0.284	0.970 ± 0.034	0.943 ± 0.054	0.262 ± 0.272
	C	**0.954 ± 0.043**	**0.915 ± 0.068**	0.260 ± 0.250	0.980 ± 0.019	0.961 ± 0.033	0.148 ± 0.143

*R* Res-UNet, *D* DeepLab v3 plus, *S* Swin-UNet, *U* UNeXt, *C* CENet

The bold values indicate the optimal values for different models at the current categories and metrics

### Agreement of IVUS quantitative measurement parameters

**Fig. 3:**
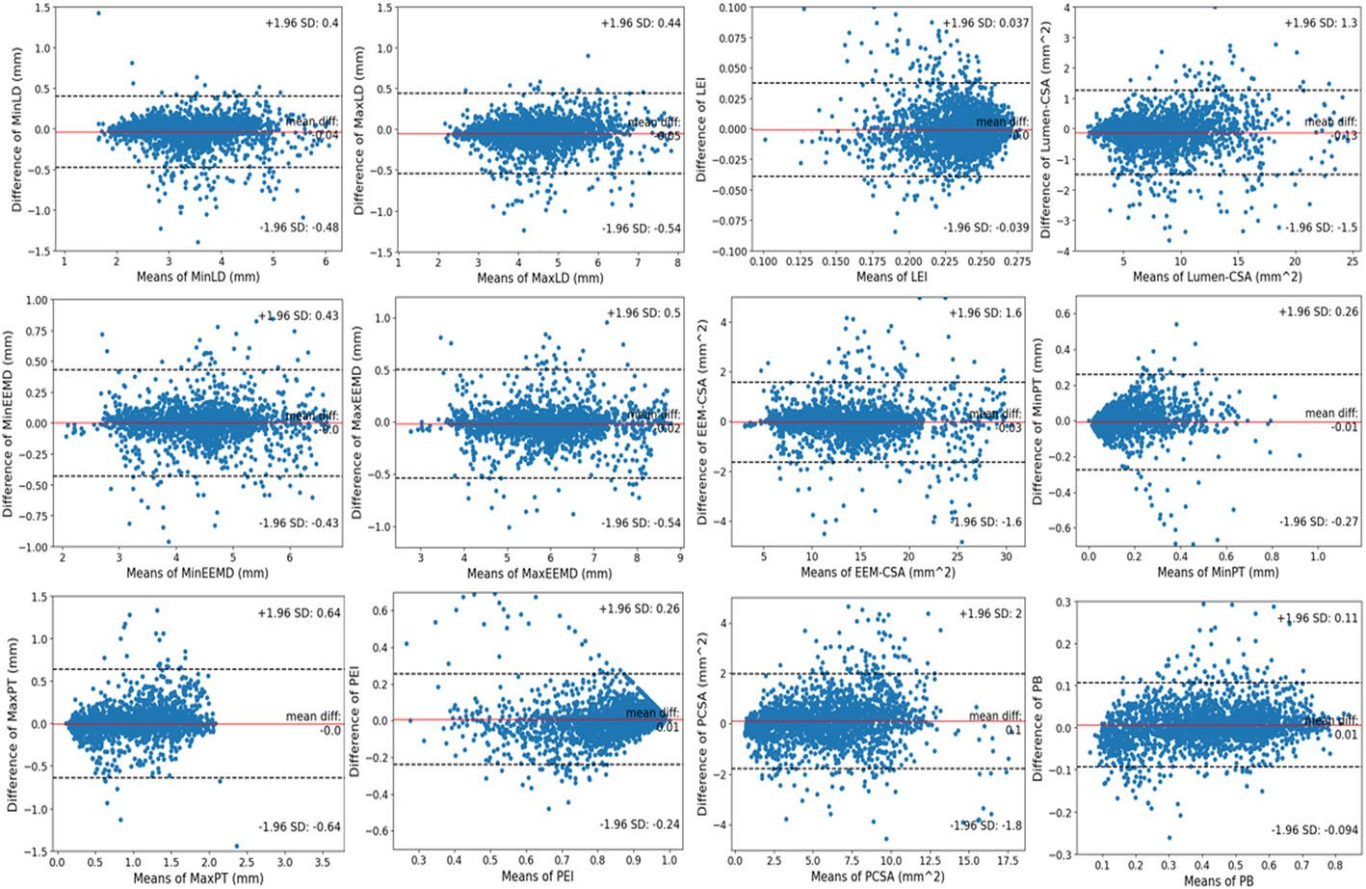
The Bland–Altman plots for the comparison of all 12 IVUS quantitative measurement parameters between model segmentations and manual measurements. *MinLD* minimum lumen diameter, *MaxLD* maximum lumen diameter, *LEI* lumen eccentricity index, *Lumen-CSA* lumen cross-sectional area, *MinEEMD* minimum EEM diameter, *MaxEEMD* maximum EEM diameter, *EEM-CSA* EEM cross-sectional area, *MinPT* minimum plaque thickness, *MaxPT* maximum plaque thickness, *PEI* plaque eccentricity index, *PCSA* plaque cross-sectional area, *PB* plaque burden

The ICC of IVUS quantitative measurement parameters between model segmentations and manual measurements is shown in Table 4. Eight of all 12 parameters had extremely strong ICC (ICC > 0.9), two had a strong consistency (0.6 < ICC < 0.8), and the remaining two had a moderate consistency (0.4 < ICC < 0.6). The Bland–Altman plot in Fig. 3 also demonstrated the extremely strong agreement between the model’s prediction and manual parameters.

**Table 4:** The ICC and 95% confidence interval (CI) for the comparison of all 12 IVUS quantitative measurement parameters between model segmentations and manual measurements

Parameter types	Parameters	ICC	95% CI
Lumen	MinLD	0.955	(0.938–0.968)
	MaxLD	0.969	(0.953–0.980)
	LEI	0.683	(0.641–0.719)
	Lumen-CSA	0.983	(0.979–0.985)
EEM	MinEEMD	0.961	(0.932–0.980)
	MaxEEMD	0.967	(0.934–0.986)
	EEM-CSA	0.986	(0.983–0.989)
Plaques	MinPT	0.539	(0.428–0.669)
	MaxPT	0.784	(0.723–0.840)
	PEI	0.532	(0.459–0.607)
	PCSA	0.951	(0.943–0.957)
	PB	0.955	(0.948–0.961)

*MinLD* minimum lumen diameter, *MaxLD* maximum lumen diameter, *LEI* lumen eccentricity index, *Lumen-CSA* lumen cross-sectional area, *MinEEMD* minimum EEM diameter, *MaxEEMD* maximum EEM diameter, *EEM-CSA* EEM cross-sectional area, *MinPT* minimum plaque thickness, *MaxPT* maximum plaque thickness, *PEI* plaque eccentricity index, *PCSA* plaque cross-sectional area, *PB* plaque burden

### Comparison with previous studies

We also compared the metrics of our best model with the metrics of previous related studies in Table 5. Our model achieved the best current segmentation performance, and obtained optimal values on all metrics with DSC, IoU and HD.

**Table 5:** Comparison of IVUS segmentation metrics between our study and previous related studies

Related studies	Lumen			EEM		
	DSC	IoU	HD (mm)	DSC	IoU	HD (mm)
Du et al. [11]	0.927	0.911	0.336	0.944	0.933	0.367
Kim et al. [12]	0.900	0.810	1.460	0.840	0.730	1.460
IVUS-Net [8]	0.947	0.900	0.260	0.925	0.860	0.480
Balocco et al. [13]	0.936	0.880	0.340	0.953	0.910	0.310
Ours	**0.958**	**0.921**	**0.237**	**0.975**	**0.951**	**0.184**

The segmentation performance of eight participants on two IVUS datasets is provided in the study by Balocco et al. [13] For comparison purposes, we selected the lumen and EEM optimal results for reference

The bold values indicate the optimal values for different studies

## Discussion

In this study, we aimed to explore the accurate segmentation performance of deep learning 2D image segmentation models on lumen and EEM under a very large IVUS image cohort dataset, as well as quantitative IVUS parameter evaluation based on segmentation. The results of the testing cohort showed that the CNN-based Res-UNet and CENet network structures have outstanding performance on IVUS image segmentation. And the quantitative IVUS parameters obtained by automatic segmentation based on the model were in excellent agreement with that calculated by manual segmentation.

The four main contributions of this work are as follows: (1) we have constructed a large image segmentation dataset of IVUS, with 11,070 images containing seven diverse IVUS image categories, and also set up a professional annotation team to ensure the quality and reliability of lumen and EEM’s masks. (2) The performance of the latest state of the art (SOTA) medical image segmentation model on a large IVUS image dataset was tested to explore the segmentation generalizations capabilities of different IVUS image classes. (3) On IVUS images, the CNN-based 2D medical image segmentation model outperformed the currently popular Transformer and MLP structure-based image segmentation models. (4) The IVUS quantitative measurement parameters calculated based on the deep learning model segmentation have excellent agreement with the manually segmented measurement parameters.

Deep learning image segmentation models have made notable progress in recent years [14–16]. In terms of model structure, it is mainly divided into CNN based, Transformer and MLP based, and the recent prompt-based structural design [17, 18]. Although current Transformer-based segmentation models have achieved outperforming CNN-based models on some segmentation tasks, Swin-UNet does not stand out as far as IVUS segmentation was concerned. UNeXt is a lightweight segmentation model based on the MLP, which can be seen to have a parametric count of only 1.47 million compared to other models, and in this respect UNeXt sacrifices some accuracy for speed. From Tables 2 and 3, the CNN-based segmentation models represented by CENet and Res-UNet outperform the Transformer structure-based segmentation models under tens of thousands of orders of magnitude of data in the IVUS domain. We speculated that this may be related to the relatively fixed structural hierarchy of the IVUS images themselves, which fits the learning characteristics of CNN.

Most of the IVUS measurement parameters predicted by our model were in excellent agreement with those obtained from manual measurements, but the performance was mediocre for some of the extension and plaque measurement parameters (LEI, PEI, MinPT and MaxPT). The reason for this may be related to the relatively irregular morphological features of the plaques themselves on IVUS images compared to the fixed hierarchy of the lumen and EEM. We also made a comparison of metrics with previous studies, a comparison that was perhaps not so fair because everyone is not in the same baseline, and the datasets and models used are different.

Our study also had some limitations. First, because of timely reasons we did not collect data from multi-centers in this study, which may affect the generalization ability of the model to some extent. Expanding this study to multi-centers is something that needs to be dealt with urgently in the future. Second, the IVUS probes frequency we used was 40 MHz, which is the current mainstream probes frequency. But it might be better to supplement the images with a 60 MHz frequency. Third, we have directly used existing medical image segmentation models rather than designing our own, which somewhat detracts from the novelty of this work.

## Conclusion

In conclusion, we explored upper limits for automatic segmentation of IVUS lumen and EEM under large image cohorts. Deep learning-based segmentation of IVUS images can achieve excellent segmentation accuracy, and IVUS measurement parameters obtained based on segmentation calculations can be further used for clinical evaluation.

## Methods

### Data and annotations

**Fig. 4:**
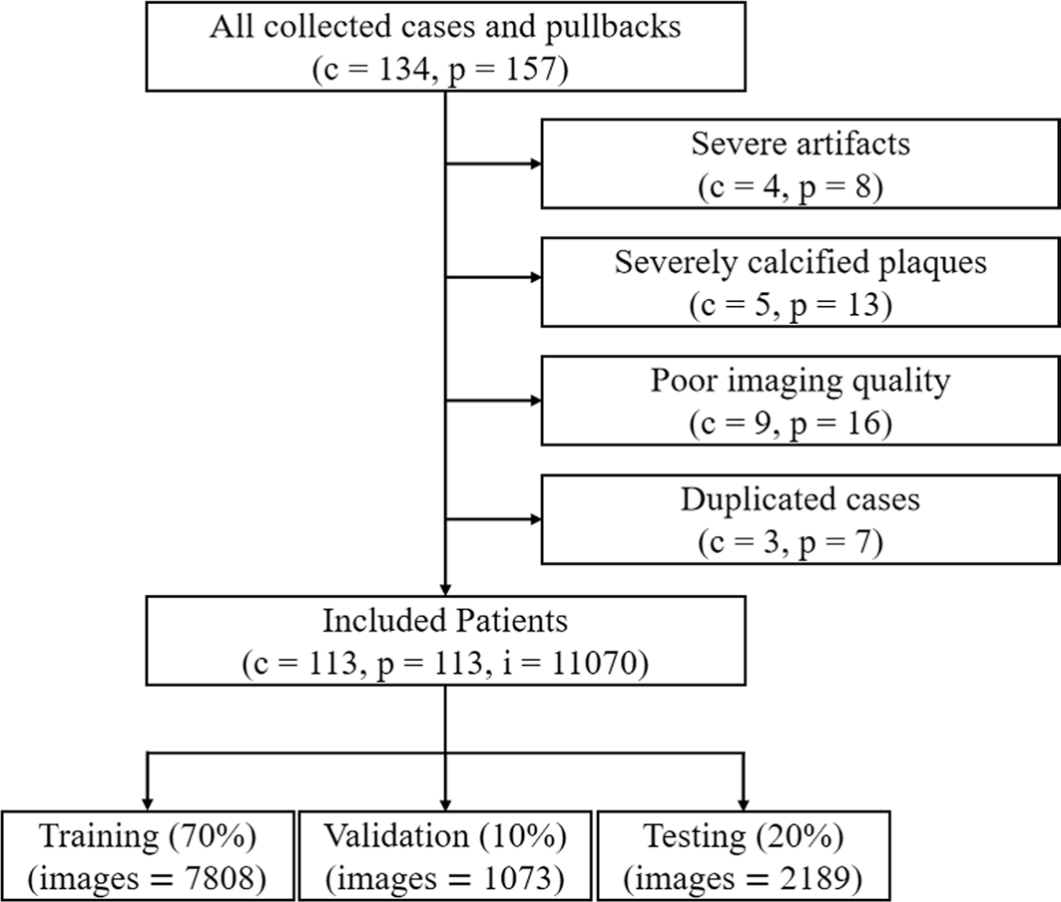
Flowchart shows patient inclusion and exclusion in deep learning segmentation. *c* cases, *p* pullbacks, *i* images

As a retrospective study, we collected 134 IVUS pullbacks from 157 patient cases at the Second Affiliated Hospital of Zhejiang University School of Medicine between December 2020 and February 2022, 97% of which had coronary artery lesions. All IVUS images were in Digital Imaging and Communications in Medicine (DICOM) format and generated by Boston Scientific’s iLab with a 40-MHz OptiCross catheter. After excluding cases with severe artifacts, severely calcified plaques, poor imaging quality and some duplicated, 113 pullbacks from 113 cases were ultimately retained. To reduce redundancy and avoid similarity between training images, we sampled about 90 to 110 images from each pullback, and the final amount of data obtained was 11,070 images. All images were anonymized and no personal patient information was involved. Patient inclusion and exclusion criteria are shown in Fig. 4.

**Fig. 5:**
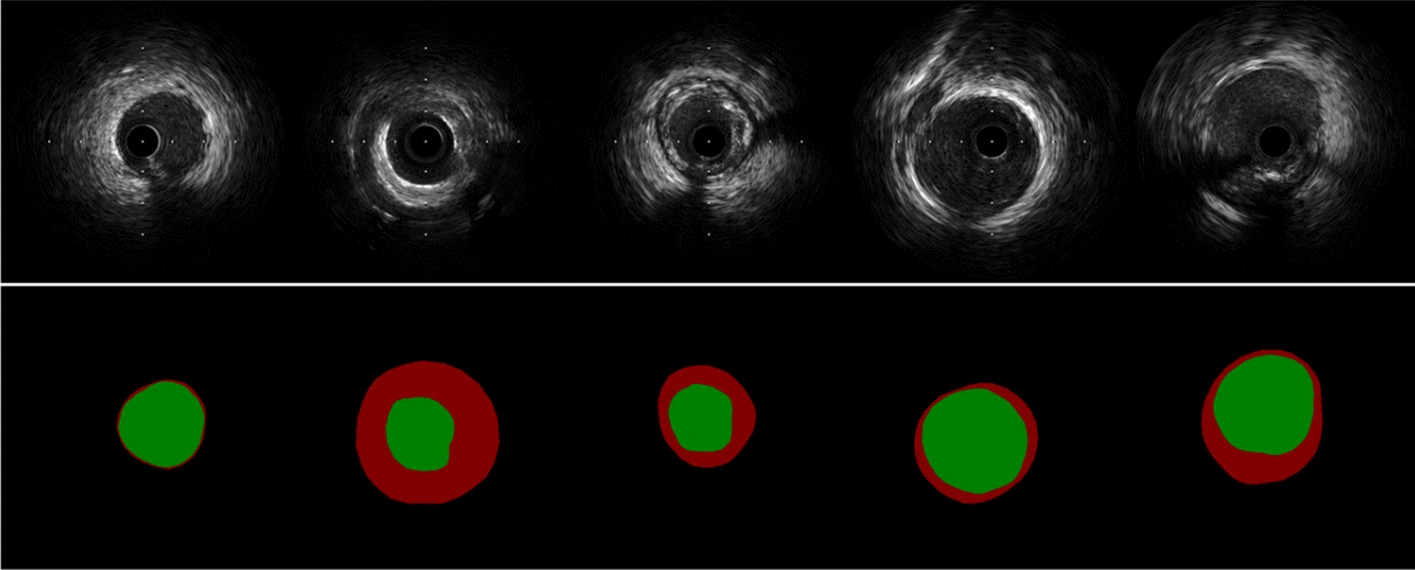
A set of 5 images containing examples of IVUS lumen and EEM annotations

The annotation requires the cardiologist to manually label the outline of the lumen and EEM and save it as a mask. To ensure the reliability and consistency of the annotation, we assembled an IVUS image annotation team including three cardiology and ultrasound specialists. Two of them with at least 5 years of experience were dedicated to IVUS annotation, and the remaining one with at least 10 years of experience was responsible for the quality review of the annotation. The flow of the annotation is as follows: the two annotators perform the IVUS contouring separately, and if the intersection over union (IoU) between the two masks is greater than 0.98, we can assume that the annotation of the two is the same. If not, a third reviewer will make the determination. The annotation tool uses Labelme version 5.1.0, an open-source image polygonal annotation software with Python. Examples of the annotations of the IVUS images are demonstrated in Fig. 5. We then divided the full dataset, with 70% used for training the segmentation model, 10% for validation and 20% for testing.

### Segmentation models

We experimented on our large cohort IVUS dataset with five classical, representative and recent network structures in medical image segmentation, namely Res-UNet, Deeplab v3 plus, Swin-UNet, UNeXt and CENet.

#### Res-UNet

Res-UNet uses a UNet encoder-decoder backbone, in combination with residual connections, atrous convolutions, pyramid scene parsing pooling, and multi-tasking inference [19]. To achieve consistent training as the depth of the network increases, the building blocks of the UNet architecture were replaced with modified residual blocks of convolutional layers [20]. For better understanding across scales, multiple parallel atrous convolutions with different dilation rates are employed within each residual building block. The pyramid scene parsing pooling layer is used to enhance the performance of the network by including background context information.

#### Deeplab v3 plus

Deeplab v3 plus is a novel encoder-decoder structure which employs Deeplab v3 as a powerful encoder module and a simple yet effective decoder module [21]. Deeplab v3 plus adapts the Xception model for the segmentation task and applies depthwise separable convolution to both atrous spatial pyramid pooling (ASPP) module and decoder module, resulting in a faster and stronger encoder-decoder with atrous convolution network [22, 23]. The atrous convolution is a powerful tool to control the resolution of features computed by deep convolutional neural networks and adjust the filter's field-of-view to capture multi-scale information, generalizing the standard convolution operation. The depthwise separable convolution factorizes a standard convolution into a depthwise convolution followed by a point-wise convolution.

#### Swin-UNet

Swin-UNet is a UNet-like pure Transformer for medical image segmentation [24]. The tokenized image patches are fed into the Transformer-based U-shaped Encoder–Decoder architecture with skip-connections for local–global semantic feature learning. Swin-UNet consists of an encoder, bottleneck, decoder, and skip connections. The encoder uses hierarchical Swin Transformer with shifted windows to extract context features [25]. The symmetric Swin Transformer-based decoder with patch expanding layer is designed to perform the up-sampling operation to restore the spatial resolution of the feature maps. Similar to the U-Net, the skip connections are used to fuse the multi-scale features from the encoder with the up-sampled features.

#### UNeXt

UNeXt is the first convolutional multilayer perceptron (MLP)-based network for image segmentation [26]. It is designed in an effective way with an early convolutional stage and an MLP stage in the latent stage. The tokenized MLP block is used to tokenize and project the convolutional features. The MLPs are used to model the representation and focus on learning local dependencies by shifting the channels of the inputs. The network also includes skip connections between various levels of encoder and decoder to fuse multi-scale features.

#### CENet

CENet is a context encoder network designed to capture more high-level information and preserve spatial information for 2D medical image segmentation [27]. It mainly consists of three major components: a feature encoder module, a context extractor, and a feature decoder module. The feature encoder module uses a pretrained ResNet block as a fixed feature extractor. The context extractor module is formed by a newly proposed dense atrous convolution (DAC) block and residual multi-kernel pooling (RMP) block. The feature decoder module is used to restore the high-level semantic features extracted from the feature encoder module and context extractor module.

### IVUS quantitative parameter measurement

The automated segmentation of the lumen and EEM from IVUS images allows us to measure a number of quantitative parameters that reflect the extent of coronary artery disease. Given a set of IVUS pullbacks, the lumen and EEM are segmented for all images and parameters including lumen measurement parameters, EEM measurement parameters and plaque measurement parameters [28]. Specifically, the lumen measurement parameters include minimum lumen diameter, maximum lumen diameter, lumen eccentricity index and lumen cross-sectional area (CSA). The EEM measurement parameters include minimum EEM diameter, maximum EEM diameter and EEM-CSA. The plaque measurement parameters include maximum plaque thickness, minimum plaque thickness, plaque eccentricity index, plaque CSA and plaque burden. Both the minimum lumen diameter and the cross-sectional area are important references for the degree of lumen stenosis.

### Statistical analysis

All statistical analyses were performed using R statistical and computing software (http://www.r-project.org). The statistical analysis of this study was reflected in three aspects. First, detailed demographic statistics and hypothesis testing were carried out on the study cohort and the divided dataset. Secondly, for the segmentation model results, dice similarity coefficient (DSC), intersection over union (IoU) and Hausdorff distance (HD) were used to measure the accuracy of the algorithm. Finally, for the IVUS quantitative measurement parameters, intraclass correlation coefficient (ICC) and Bland–Altman analysis were performed to analyses the agreement of the automated segmentation measurements with the manual segmentation measurements [29].

## Data Availability

The datasets analyzed during the current study are available from the corresponding author on reasonable request.

## Abbreviations

IVUS: Intravascular ultrasound
EEM: External elastic membrane
FCN: Fully convolutional network
CAD: Coronary artery disease
LAD: Left anterior descending
LCX: Left circumflex
RCA: Right coronary artery
ANOVA: Analysis of variance
DSC: Dice similarity coefficients
IoU: Intersection over union
HD: Hausdorff distance
MinLD: Minimum lumen diameter
MaxLD: Maximum lumen diameter
LEI: Lumen eccentricity index
Lumen-CSA: Lumen cross-sectional area
MinEEMD: Minimum EEM diameter
MaxEEMD: Maximum EEM diameter
EEM-CSA: EEM cross-sectional area
MinPT: Minimum plaque thickness
MaxPT: Maximum plaque thickness
PEI: Plaque eccentricity index
PCSA: Plaque cross-sectional area
PB: Plaque burden
SOTA: State of the art
ICC: Intraclass correlation coefficient
MLP: Multilayer perceptron
ASPP: Atrous spatial pyramid pooling
DAC: Dense atrous convolution
RMP: Residual multi-kernel pooling
